# A 23-Year Comprehensive Analysis of over 4000 Liver Transplants in Türkiye: Integrating Clinical Outcomes with Public Health Insights

**DOI:** 10.3390/healthcare14020163

**Published:** 2026-01-08

**Authors:** Deniz Yavuz Baskiran, Sezai Yilmaz

**Affiliations:** Liver Transplantation Institute, İnönü University, Malatya 44210, Türkiye

**Keywords:** liver transplantation, epidemiology, living donor, disease patterns

## Abstract

**Highlights:**

**What are the main findings?**

- This 23-year single center analysis of 4011 liver transplants demonstrates that over 86% of procedures were living donor liver transplants, positioning the center among the highest-volume LDLT programs globally.
- Distinct and clinically relevant age dependent etiological patterns were identified: infectious causes dominated adults, whereas toxic etiologies, bile duct diseases, and metabolic disorders were predominant in pediatric recipients.

**What are the implications of the main findings?**

- The high living donor rate highlights the need for strengthening donor safety policies, regional public health planning, and organ donation awareness programs across Türkiye.
- The age specific etiological differences can guide targeted prevention, early diagnosis, and optimized allocation of healthcare resources for diverse patient groups

**Abstract:**

**Background:** This study seeks to evaluate the 23 year experience of the İnonu University Liver Transplantation Institute from a public health perspective by examining demographic patterns, etiological factors, and transplantation trends between 2002 and 2025. **Aims:** This analysis aims to provide insights into the epidemiological landscape of liver transplantation in Türkiye from a public health perspective. **Methods:** In this retrospective cross sectional study, we analyzed 4011 liver transplant procedures performed between March 2002 and March 2025. Recipient demographics, disease etiologies, donor characteristics, and patients geographic distribution were assessed to delineate regional health needs and service utilization patterns. **Results:** A total of 4011 patients were included. The cohort comprised 2618 males (65.3%) and 1393 females (34.7%). Recipients were classified as adult (*n* = 3232, 80.9%) or pediatric (*n* = 779, 19.1%). Among adults, infectious etiologies were the most prevalent (35.5%), followed by cryptogenic liver cirrhosis (24.7%). In contrast, pediatric patients most commonly presented with toxic etiologies (29.4%), metabolic disorders (22.6%) and bile duct diseases (15.9%). Most liver transplantations were performed using living donors (*n* = 3481, 86.8%), while deceased donors accounted for 530 procedures (13.2%). Additionally, 244 living donor liver transplantations were performed via liver paired exchange (LPE). **Conclusions:** These findings may inform resource allocation, health policy development, and the optimization of transplantation services. This center-based model offers a useful framework for characterizing regional health needs and strengthening community health, particularly through prevention, screening, and early intervention strategies for liver diseases.

## 1. Introduction

Liver transplantation (LT) is considered the only curative treatment option for end-stage liver failure, hepatocellular carcinoma, and some primary hepatobiliary diseases [1]. The success of this treatment method has significantly increased over the last 30 years due to advances in surgical techniques, improvements in immunosuppressive treatment options, and optimization of perioperative care processes [2].

LT practices in Türkiye have gained remarkable momentum since the early 2000s; not only in terms of the number of transplants but also in parameters such as center infrastructure, multidisciplinary team coordination, and pediatric transplantation experience, becoming competitive at the international level. Türkiye is one of the few countries worldwide that is a leader in living donor liver transplantation (LDLT). Approximately 1500–1800 LTs are performed annually in the country, with more than 80% coming from living donors [3,4]. This situation highlights the importance of social solidarity brought about by strong family ties and extensive kinship relationships, despite the low rates of deceased donation in Türkiye.

The Liver Transplantation Institute of İnonu University is one of the strongest representatives of this development in our country. The center has shown consistent growth and development in its LT journey that began in 2002 becoming a regional and international reference center. The center, which has performed 4011 liver transplants over a 23-year period, is Türkiye’s leading LT center, with annual transplant numbers exceeding 300 in some years. The Liver Transplantation Institute of İnonu University stands out not only for its surgical achievements but also for its contributions to analyzing and meeting regional health needs in terms of public health and health policies and improving the health status of the community. Furthermore, understanding the mechanistic pathways shared across different disease etiologies provides essential context for interpreting epidemiological patterns and may help guide future directions in transplant-related research.

This study aims to evaluate the 23 year experience of the institute in light of epidemiological data, highlighting the importance of the center based model in health service planning. The findings are intended to contribute to both local health planning and the direction of national policies.

## 2. Materials and Methods

### 2.1. Study Design and Setting

This retrospective, cross-sectional and descriptive study was conducted using data from liver transplantations (LTs) performed at the institute between 1 March 2002, and 31 March 2025. This study was approved by the İnonu University Non Interventional Clinical Research Ethics Committee (Approval No: 2025/7590) in Malatya, Türkiye (https://etik.inonu.edu.tr/panel/index, accessed on 4 January 2026). Given the retrospective design, the requirement for informed consent was waived by the ethics committee. All data were anonymized and used exclusively for scientific purposes.

### 2.2. Study Population

All patients who underwent LT during the study period and had complete baseline demographic and clinical data were included. Patients who were listed for transplantation but did not undergo the procedure, or those with missing essential demographic information, were excluded from the analysis. Pediatric recipients were defined as individuals younger than 18 years. Donor type was classified as living or deceased according to the procurement source. Kinship relationships were categorized as first degree relatives (parent, sibling, spouse) or second-degree relatives and beyond.

### 2.3. Data Collection

Data were obtained from the hospital’s digital health information management system and internal archival records. The dataset comprised demographic characteristics of both donors and recipients (age, sex, hometown, and blood type), transplantation type (living vs. deceased), date of transplantation, and donor–recipient relationship. Prior to analysis, all data were reviewed and preprocessed to ensure consistency and suitability for statistical evaluation.

### 2.4. Statistical Analysis

All statistical analyses were performed using SPSS software version 25.00 (IBM Corp., Armonk, NY, USA). Descriptive statistics were used to summarize the demographic and clinical variables. Continuous variables were expressed as mean ± standard deviation (SD) or median with range, depending on distribution normality. Categorical variables were presented as frequencies and percentages.

Comparisons between groups were performed using the independent samples *t*-test for normally distributed continuous variables and the Mann–Whitney U test for non normally distributed variables. The chi-square test was used to evaluate associations between categorical variables. A *p*-value < 0.05 was considered statistically significant.

## 3. Results

### 3.1. Patient Demographics and Donor Characteristics

In this study, a total of 4011 patients were analyzed. The cohort consisted of 2618 males (65.3%) and 1393 females (34.7%) with a mean age of 40.3 ± 20.6 years (median 47, range 0–75). As shown in Table 1, the study population was divided into adult (*n* = 3.232, 80.6%) and pediatric (*n* = 779, 19.4%) recipients. Adult recipients had a mean age of 48.5 ± 13.2 years with a higher pro-portion of males (67.72%), while pediatric recipients had a mean age of 6.5 ± 5.5 years with a more balanced gender distribution (55.1% male, 44.9% female).

The majority of liver transplantations were performed with living donors (*n* = 3.237, 80.70%), followed by deceased donors (*n* = 530, 13.2%) and donors that obtained with the LPE (Liver pare Exchange) system (*n* = 244, 6.1%). When examining donor characteristics, the mean age of donors was 31.9 ± 11.0 years (median 30, range 0–76), with 59.9% being male and 40.1% female. Among the 3481 living donors, the most common donor relationship was son (21.4%), followed by sibling (16.8%) and daughter (12.2%).

### 3.2. Etiology of Liver Disease in Adult and Pediatric Patients

**Figure 1:**
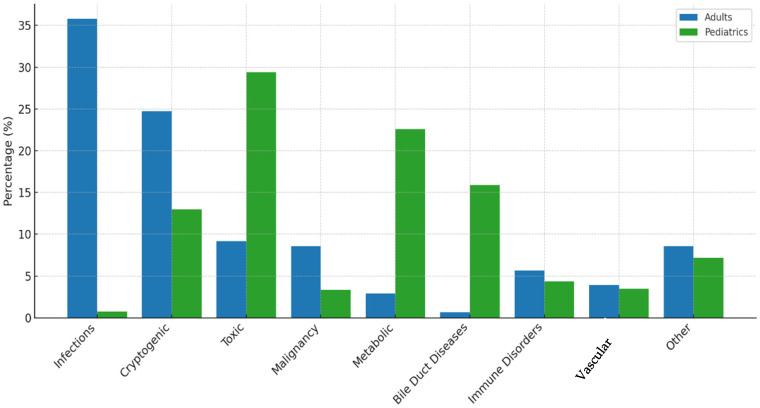
Comparison of Liver Disease Etiologies Between Adult and Pediatric Transplant Patients.

The etiologies of liver diseases requiring transplantation differed significantly between adult and pediatric populations (Table 2 and Table 3, Figure 1). In adults, infectious etiologies were most prevalent (35.77%), followed by cryptogenic liver cirrhosis (24.7%), toxic causes (9.2%), and malignancies (8.6%). In contrast, pediatric patients most commonly presented with toxic etiologies (29.4%), metabolic disorders (22.6%) and bile duct diseases (15.9%).

**Figure 2:**
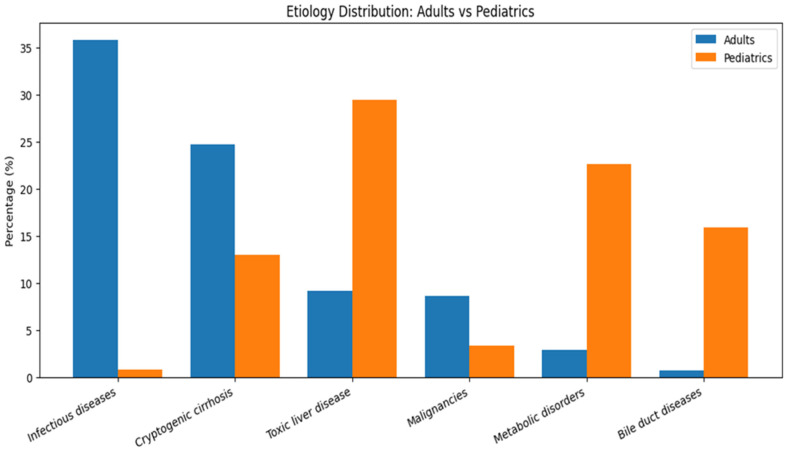
Etiology Distrubition: adult and Pediatrics.

Statistical analysis demonstrated significant differences (*p* < 0.0001) between adult and pediatric populations for infectious diseases (35.8% vs. 0.8%), cryptogenic cirrhosis (24.7% vs. 13%), toxic liver disease (9.2% vs. 29.4%), malignancies (8.6% vs. 3.3%), metabolic disorders (2.9% vs. 22.6%), and bile duct diseases (0.7% vs. 15.9%). No significant differences were observed for immune-mediated disorders (*p* = 0.2694), vascular etiology (*p* = 0.6192), and other etiologies (*p* = 0.4044) (Figure 2).

### 3.3. Age-Specific Patterns of Liver Disease

**Figure 3:**
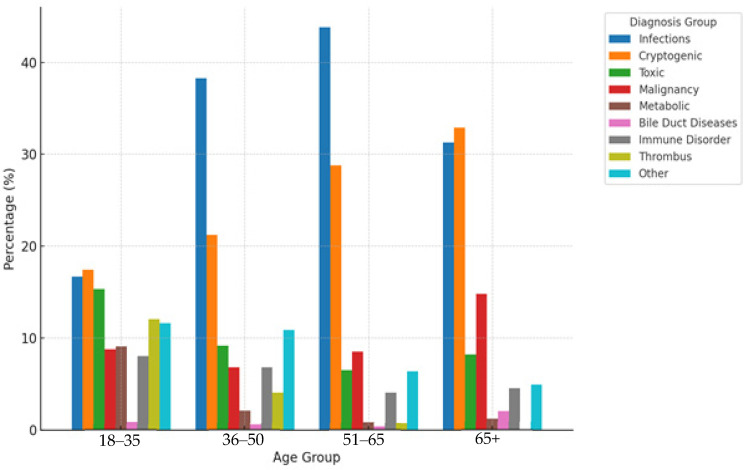
Diagnostic Patterns by Age Group in Adult Liver Transplant Patients.

When analyzing adult recipients by age groups (Table 4 and Table 5, Figure 3), significant variations in disease etiology were observed. Infectious causes showed a marked increase from young adults (16.69% in 18–35 years) to middle-aged adults (43.8% in 51–65 years), with odds ratios of 3.10 (*p* = 0.009) for 36–50 vs. 18–35 and 3.89 (*p* = 0.007) for 51–65 vs. 18–35. Cryptogenic liver disease also demonstrated an age-dependent increase, reaching 32.9% in patients over 65 years. In contrast, toxic liver disease showed a significant decline with age, from 15.4% in 18–35 years to 6.5% in 51–65 years. Metabolic disorders were predominantly observed in younger adults (9.1% in 18–35 years) compared to older age groups (0.8% in 51–65 years), with statistically significant differences (*p* < 0.001). Vascular problems were particularly prevalent in the youngest adult group (12.07% in 18–35 years) and virtually absent in those over 65 years (0%, *p* < 0.001).

**Figure 4:**
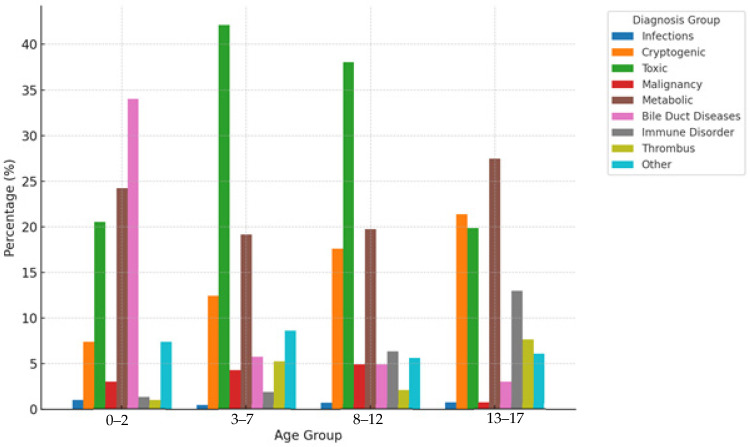
Diagnostic Patterns by Age Group in Pediatric Liver Transplant Patients.

Similarly, pediatric recipients exhibited distinct age-related patterns (Table 6 and Table 7, Figure 4). Cryptogenic liver disease increased significantly with age, from 7.4% in 0–2 years to 21.4% in 13–17 years (OR = 3.40, *p* = 0.001). Toxic liver disease was most common in intermediate age groups (42.1% in 3–7 years and 38% in 8–12 years). Bile duct diseases showed a dramatic decline with age, from 34% in 0–2 years to 3% in 13–17 years (*p* < 0.001). Immune-mediated disorders demonstrated a significant age-dependent increase, from 1.4% in 0–2 years to 13% in 13–17 years (OR = 10.92, *p* = 0.001).

### 3.4. Blood Group Analysis

Blood group distribution analysis showed that the most common blood type among recipients was A+ (34.6%), followed by O+ (21.23%), B+ (17.9%), and AB+ (16.8%). Among donors, O+ (35.3%) was most common, followed by A+ (26.3%), B+ (12.4%), and A− (7.3%). The cross-tabulation of donor and recipient blood groups demonstrated the expected compatibility patterns, with ABO-identical transplantations being most frequent (Table 8 and Table 9).

### 3.5. Donor-Recipient Relationships and Geographic Patterns

Analysis of donor-recipient relationships revealed significant differences between pediatric and adult recipients (Table 10, Table 11 and Table 12). For pediatric recipients, parents were the predominant donors (mother: 24%, father: 16.2%), followed by deceased donors (20.1%). In contrast, adult recipients most commonly received organs from their children (sons: 23.2%, daughters: 13.3%), siblings (16.7%), and deceased donors (11.7%). These differences were statistically significant (*p* < 0.05 to *p* < 0.001).

Regarding geographic distribution, the majority of recipients originated from eastern and southeastern regions of Turkey (Table 13). The top five provinces for all recipients were Malatya (8.1%), Şanlıurfa (8.03%), Diyarbakır (7.8%), foreign nationals primarily from Syria (7.6%). Notably, there were differences in geographic distribution between pediatric and adult recipients, with Syrian refugees constituting 20.2% of pediatric recipients but only 4.5% of adult recipients. Deceased donors were predominantly from western and Mediterranean regions (Table 14).

### 3.6. Yearly Distribution of Liver Transplantations

**Figure 5:**
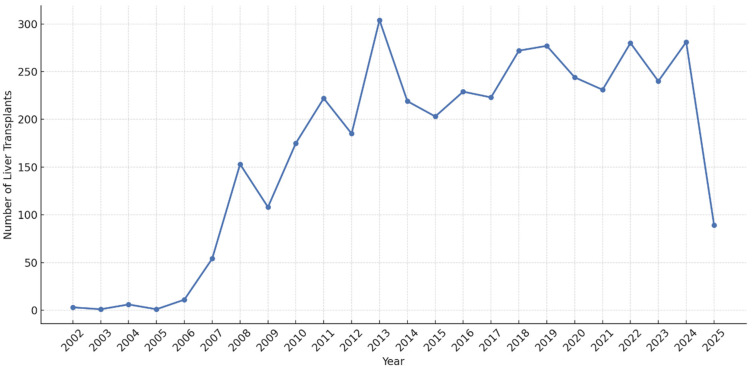
The Annual number of liver transplantation.

The annual number of liver transplantations performed at the institute exhibited a notable progression from 2002 to 2025 (Table 15). The annual number of liver transplantation (Figure 5). The program began modestly with only 3 transplantations in 2002, growing steadily to reach 11 in 2006. A significant increase was observed in 2007 (*n* = 54), followed by substantial growth in 2008 (*n* = 153). The program continued to expand, with annual transplantation numbers reaching over 200 cases in 2011 (*n* = 222) and peaking at 304 cases in 2013, making it one of the most productive years in the center’s history.

The annual transplantation volume has remained consistently high in recent years, with 272 cases in 2018, 277 in 2019, 244 in 2020, 231 in 2021, 280 in 2022, 240 in 2023, and 281 in 2024. Notably, despite the global COVID-19 pandemic in 2020–2021, the center maintained robust transplantation numbers, with only a modest decrease from pre-pandemic volumes (244 in 2020 and 231 in 2021 compared to 277 in 2019). The data for 2025 includes only the first quarter (*n* = 89), projecting a similar annual volume to previous years.

This consistent growth and maintenance of high transplantation volumes reflect the center’s increasing capacity, expertise, and regional significance over the 23-year period analyzed.

## 4. Discussion

Our analysis of 4011 liver transplantations performed at the institute over a 23-year period provides substantial insights into the epidemiological patterns, etiological factors, and demographic characteristics of patients requiring liver transplantation in Türkiye. The findings highlight the unique aspects of liver transplantation practice in the country, particularly the predominance of living donor transplantation and regional variations in disease etiology.

Our etiological spectrum shows distinct regional characteristics when compared to major international registries. While our adult cohort demonstrates infectious causes as the leading indication at 36%, predominantly hepatitis B and C, this contrasts sharply with contemporary North American patterns where NAFLD and NASH account for 32–38% of transplants, alcoholic liver disease for 22–25%, and hepatitis C for 15–18% according to recent UNOS data [5,6]. European patterns from ELTR registries show alcoholic liver disease leading at 28–32%, followed by NAFLD at 20–25% and hepatitis C at 12–15% [7,8]. Asian patterns more closely resemble our findings, with Japan and South Korea showing viral hepatitis predominance at 40–45%, though both countries demonstrate declining trends following decades of universal vaccination programs [9,10]. This divergence reflects Turkey’s ongoing epidemiological transition where despite universal hepatitis B vaccination implemented in 1998, our current transplant population with a mean age of 40 years predominantly comprises individuals born before vaccination implementation [11]. Regional hepatitis B prevalence remains heterogeneous across Turkey, ranging from 2–8% across different regions, which explains the persistent dominance of viral hepatitis in transplant indications [12]. In pediatric populations, our toxic etiology predominance at 30%, primarily from accidental poisonings, substantially exceeds international rates of 12–15% in North America and 8–12% in Europe [13,14], representing a critical preventable public health target unique to our regional setting.

Age-stratified analysis revealed distinct and clinically meaningful patterns across both adult and pediatric populations. In adult recipients, infectious etiologies demonstrated a progressive increase with age, rising from 17% in the 18–35-year age group to 44% in the 51–65-year age group with an odds ratio of 3.89. This age-dependent pattern reflects cumulative hepatitis exposure over decades, particularly in cohorts born before widespread vaccination programs [15]. Conversely, metabolic disorders showed marked concentration in younger adults at 9% compared to only 1% in older age groups, emphasizing the critical value of newborn screening programs that enable early intervention and potentially prevent progression to end-stage liver disease [16,17]. Cryptogenic liver disease demonstrated an age-dependent increase, reaching 33% in patients over 65 years. In pediatric populations, immune-mediated disorders exhibited a dramatic increase with age, from 1% in children aged 0–2 years to 13% in adolescents aged 13–17 years with an odds ratio of 10.92, aligning with the typical onset patterns of autoimmune liver diseases during adolescence [18,19]. Toxic liver disease showed peak prevalence in intermediate age groups at 42% in children aged 3–7 years, while bile duct diseases demonstrated a dramatic decline with age from 34% in infants to 3% in adolescents [20].

Our living donor liver transplantation rate of 87% positions this center among the highest-volume programs globally, comparable only to select Asian centers where South Korea achieves 85–90%, Japan 80–85%, and select centers in India 75–85% [21,22,23]. This stands in stark contrast to Western transplantation systems where North America reports less than 5% living donor procedures, Europe less than 10%, and Australia less than 15% [24,25]. This pattern reflects multiple interconnected factors including persistently low deceased donor rates in Turkey at 8–10 per million population compared to 25–30 per million in Spain and 20–25 per million in the United States [26,27], strong familial support systems deeply embedded in Turkish culture, and widespread religious and cultural acceptance of living donation among family members. However, this living donor-predominant model creates distinct challenges for healthcare systems that are not encountered in deceased-donor-predominant programs, particularly regarding long-term donor safety monitoring, comprehensive lifetime follow-up protocols, and appropriate allocation of healthcare resources to ensure donor welfare. The 244 liver paired exchange procedures performed at our center demonstrate operational sophistication and program maturity comparable to leading Asian transplant centers.

Chronic liver diseases of diverse etiologies often converge on oxidative stress-mediated injury and fibrosis. Oxidative stress plays a central pathogenic role in the progression of viral hepatitis, non-alcoholic fatty liver disease (NAFLD), alcoholic liver disease, and drug-induced liver injury [27,28]. Circulating and tissue oxidative stress biomarkers, including lipid peroxidation products, protein adducts, and DNA oxidation markers, have been increasingly studied as tools to understand disease progression, predict transplant outcomes, and stratify risk in patients with end-stage liver disease [29,30]. Understanding the mechanistic underpinnings shared across different etiologies provides important context for interpreting epidemiological patterns and may inform future directions in transplant-related research.

Donor-recipient relationships reflect traditional Turkish family structures and kinship patterns. In adult transplantation, children served as predominant donors with sons donating in 23% of cases and daughters in 13%, while parents donated primarily to pediatric recipients with mothers comprising 24% and fathers 16% of donations. Spousal donation comprised 11% of cases, notably lower than rates reported in some Western centers, which may reflect both cultural norms regarding spousal donation and medical considerations related to consanguinity patterns that remain prevalent in certain Turkish regions [31,32,33]. Geographic analysis revealed critical healthcare disparities requiring systematic policy intervention, with 65% of recipients originating from eastern and southeastern provinces while 78% of deceased donors came from western and Mediterranean regions. This spatial mismatch reflects complex interplay of infrastructure development gaps, socioeconomic disparities, and substantial regional variations in organ donation awareness and acceptance [34,35].

Our regional findings translate into five specific policy priorities that connect epidemiological patterns directly to actionable health interventions. First, the predominance of viral hepatitis as the indication for 36% of adult transplants necessitates targeted hepatitis control measures including expanded catch-up vaccination programs specifically designed for adults born before 1998 who represent the current transplant-age cohort, implementation of targeted screening programs in Eastern and Southeastern regions where recipient density is highest, strengthened antiviral treatment accessibility to prevent cirrhosis progression, and development of regional hepatitis elimination roadmaps aligned with WHO 2030 targets [36,37]. Economic modeling suggests each prevented cirrhosis case saves an estimated 180,000 to 250,000 US dollars in transplant-related healthcare costs, providing strong justification for prevention investment [38]. Second, pediatric poisoning prevention emerges as a critical public health priority given that 30% of pediatric transplants resulted from toxic causes with peak incidence of 42% in children aged 3–7 years. Evidence-based interventions should include mandatory child-resistant packaging regulations for all hepatotoxic medications and household chemicals, implementation of a national parental education campaign coordinated through preschool enrollment systems to reach families during the highest-risk developmental period, expansion of poison control center capacity particularly in Eastern provinces where access currently remains limited, and enhancement of pediatric intensive care unit infrastructure for acute liver failure management [39,40]. A realistic target would achieve 50% reduction in toxic-related pediatric liver transplants within five years, potentially sparing 20–25 children annually from transplantation and its lifelong medical implications. Third, addressing the 13% deceased donor rate and 87% living donor dependence requires multi-faceted organ donation awareness initiatives. Policy recommendations include intensified national public awareness campaigns particularly targeting Western and Mediterranean regions that currently serve as primary deceased donor sources, consideration of opt-out consent system implementation following Spain’s successful model that achieves 46 donors per million population [41], strengthening of hospital-based donor coordination programs with dedicated professional coordinators, and creation of regional donor coordination centers in high-population areas to improve procurement infrastructure [42,43]. The achievable goal should target increasing deceased donor rates to 15–20 per million population, which would substantially reduce living donor dependency while maintaining program volume. Fourth, the large volume of 3481 living donors including 244 paired exchange procedures necessitates comprehensive living donor safety frameworks that match international standards. Essential components include establishment of a mandatory national living donor registry with standardized lifetime follow-up protocols, implementation of uniform donor selection criteria and informed consent procedures across all transplant centers, creation of independent donor advocate programs to ensure donors receive unbiased information and counseling, development of long-term complication surveillance systems with public reporting of outcomes, and guarantee of universal healthcare coverage for all donor-related complications regardless of time since donation [44,45,46]. International standards mandate zero donor mortality and major complication rates below 5%, which should serve as minimum acceptable benchmarks [47]. Fifth, regional health resource allocation must address the geographic mismatch where the majority of recipients originate from Eastern and Southeastern regions while transplant infrastructure remains concentrated in Western Turkey. Strategic interventions should include development of satellite transplant programs in underserved regions to improve access and reduce travel burden on families, establishment of regional liver disease screening and early intervention programs that enable diagnosis before decompensated cirrhosis develops, creation of telemedicine consultation networks linking regional hospitals to transplant centers for expert guidance, and targeted workforce development initiatives for hepatology and transplant surgery in Eastern provinces where specialist shortages currently limit local capacity [48,49]. Expected outcomes include earlier diagnosis enabling better pre-transplant optimization, reduced geographic disparities in access to care, and more equitable distribution of healthcare resources across regions.

Our center’s sustained high-volume living donor liver transplantation program over 23 years demonstrates operational maturity, technical expertise, and regional significance comparable to leading international centers [50,51]. The consistent annual transplant volumes, successful implementation of complex procedures including liver paired exchange, and comprehensive outcomes tracking provide valuable epidemiological insights that extend beyond clinical metrics to inform public health policy and resource allocation decisions. However, continued program growth must be carefully balanced with systematic donor safety monitoring, rigorous outcome tracking, and strategic efforts to expand equitable access to underserved populations and regions.

This study has several limitations that warrant consideration. The single-center retrospective design may limit generalizability to other Turkish centers or international settings, though our high volume and geographic diversity of patients provide substantial representation of national patterns. Selection bias may exist as our center serves as a regional referral center, potentially attracting more complex cases or patients from specific geographic areas. Long-term outcome data beyond transplantation were not included in this analysis, precluding assessment of patient and graft survival, long-term donor complications, or quality of life outcomes. Additionally, detailed socioeconomic data, education levels, and specific household risk factors for pediatric poisonings were not systematically captured, limiting our ability to identify high-risk populations for targeted interventions. Despite these limitations, the comprehensive 23-year dataset encompassing over 4000 transplants provides robust epidemiological insights that meaningfully inform clinical practice, public health policy, and future research directions in liver transplantation.

## 5. Conclusions

In this 23-year, single-center cohort of 4011 liver transplantations, we provide a comprehensive public health–oriented snapshot of liver transplantation practice in Türkiye within a predominantly living-donor system. The very high LDLT rate and the sustained implementation of liver paired exchange procedures reflect a mature, high-volume operational model that differs meaningfully from many Western settings. Beyond describing activity and etiologic distributions, our findings underscore practical priorities for health planning: strengthening prevention and early detection strategies for viral/infectious liver diseases among adults, and implementing targeted, prevention-focused actions to reduce toxic etiologies in pediatric recipients. These data also support the need for system-level approaches that align donor-safety monitoring, referral pathways, and regional resource allocation with the realities of living-donor–dominant transplantation. Overall, this center-based experience offers policy-relevant evidence to inform prevention programs and service optimization, while also providing a framework for future multicenter analyses to further refine national planning.

## Figures and Tables

**Table 1:** Baseline Demographic Characteristics of Liver Transplant Recipients and Donor Types.

		Number ± SD	% or Median [Range]
Gender (M/F, %)		2618/1393	65.27%/34.73%
General Age		40.3 ± 20.6	47 [0–75]
Adult/Pediatric		3232/779	80.58%/19.42%
Adult Age		48.5 ± 13.2	51 [18–75]
Adult Gender (M/F, %)		2189/1043	67.72%/32.28%
Pediatric Age		6.5 ± 5.5	5 [0–17]
Pediatric Gender (M/F,%)		429/350	55.07–44.93%
Type	Living Donor	3237	80.70%
	Decased	530	13.21%
	LPE	244	6.09%

**Table 2:** Distribution of Liver Disease Etiologies Among Adult and Pediatric Transplant Recipients.

Diagnostic Groups		Diagnosis	General	Adult	Pediatric
Infections	Virutic	HBV	840 (20.94)	838 (26.12)	2 (0.26)
		HBV + HDV	152 (3.79)	153 (4.74)	0 (0)
		HCV	153 (3.81)	153 (4.74)	0 (0)
		HBV + HCV	7 (0.17)	7 (0.22)	0 (0)
	Parasitic	Echinococcus Alveolaris	28 (0.7)	27 (0.19)	1 (0.13)
		Hydatid Cyst	2 (0.04)	1 (0.03)	1 (0.13)
		Schistosomiasis	1 (0.02)	1 (0)	0 (0)
Cryptogenic	Etiology Not Found	Cryptogenic Liver Cirrhosis	900 (22.44)	799 (24.91)	101 (12.97)
Toxic	Acute	Acute Fulminant Failure	406 (10.12)	179 (5.58)	227 (29.14)
	Chronic	Ethanol Dependent	120 (2.99)	120 (3.68)	0 (0)
Malignancy	HCC	HCC	156 (3.88)	152 (5.32)	8 (1.04)
		HCC (HBV + HDV)	46 (1.14)	46 (1.43)	0 (0)
		HCC (HCV)	35 (0.87)	35 (1.1)	0 (0)
		HCC (HBV)	17 (0.42)	17 (0.53)	0 (0)
		HCC (HBV + HCV)	4 (0.1)	4 (0.13)	0 (0)
		HCC (HBV + HDV + HCV)	1 (0.02)	1 (0.03)	0 (0)
		Fibrolamellar HCC	1 (0.02)	1 (0.03)	0 (0)
	Other	Hepatoblastoma	19 (0.45)	1 (0.04)	18 (2.33)
		Cholangio Ca	10 (0.25)	10 (0.31)	0 (0)
		Hemangioendothelioma	4 (0.1)	4 (0.13)	0 (0)
		Neuroendocrine TM	7 (0.17)	7 (0.22)	0 (0)
Metabolic	Copper Metabolism	Wilson’s Disease	122 (3.03)	70 (2.18)	52 (6.42)
	Bile Acid and Transport Disorders	PFIC (All types)	34 (0.85)	0 (0.0)	34 (4.62)
	Disorders of Bilirubin Metabolism	Neonatal Cholestasis	32 (0.79)	0 (0.0)	32 (4.11)
		Crigler-Najjar Syndrome	11 (0.27)	1 (0.03)	10 (1.28)
		Dubin Johnson	1 (0.02)	0 (0.0)	1 (0.13)
	Amino Acid Metabolism Disorders	Trisonemia	11 (0.27)	0 (0.0)	11 (1.41)
		MSUD (All types)	6 (0.15)	0 (0.0)	6 (0.77)
	Protein Catabolism	Alpha 1 Antitrypsin Deficiency	3 (0.07)	1 (0.03)	2 (0.26)
	Carbohydrate Metabolism Disorders	Glycogen Storage Diseases	9 (0.22)	2 (0.06)	7 (0.9)
	Iron Metabolism Disorders	Hemochromatosis	8 (0.2)	5 (0.16)	3 (0.39)
	Oxalate Metabolism	Primary hyperoxaluria	2 (0.04)	0 (0.0)	2 (0.26)
	Steatosis	NASH	13 (0.32)	13 (0.4)	0 (0)
		Familial Hypercholesterolemia	9 (0.22)	2 (0.06)	7 (0.9)
	Heterogeneous	Other (Niemann-Pick, Cystic-Fibrosis Citrullinemia, Berardinelli-Seip syndrome. Protoporphyria, etc.)	11 (0.27)	0 (0.0)	11 (1.41)
Bile Duct Diseases	Congenital and Acquired	Biliary Atresia	112 (2.79)	0 (0.0)	112 (14.38)
		Caroli’s Disease	16 (0.4)	6 (0.19)	10 (1.28)
		Choledochal Cysts	3 (0.06)	2 (0.06)	1 (0.13)
		Primary Sclerosing Cholangitis	14 (0.35)	13 (0.41)	1 (0.13)
Immunity Disorder	Autoimmune	Autoimmune Hepatitis	217 (5.41)	183 (5.7)	34 (4.36)
Vascular	Venous	Budd-Chiari	154 (3.84)	127 (3.96)	27 (3.47)
Other	Heterogeneous (Including those with no recorded diagnosis)	Other	314 (8.20)	251(8.18)	58(7.19)

**Table 3:** Comparison of Major Diagnostic Categories Between Adult and Pediatric Liver Transplant Recipients.

Diagnostic Groups	Total	Adult	Pediatric	*p* Value
Infections	1162 (28.97%)	1156 (35.77%)	6 (0.77%)	<0.001
Cryptogenic	900 (22.44%)	799 (24.72%)	101 (12.97%)	<0.001
Toxic	526 (13.11%)	297 (9.19%)	229 (29.4%)	<0.001
Malignancy	304 (7.58%)	278 (8.6%)	26 (3.34%)	<0.001
Metabolic	270 (6.73%)	94 (2.91%)	176 (22.59%)	<0.001
Bile Duct Diseases	145 (3.62%)	21 (0.65%)	124 (15.92%)	<0.001
Immune Disorder	217 (5.41%)	183 (5.66%)	34 (4.36%)	0.2694
Vascular	154 (3.84%)	127 (3.93%)	27 (3.47%)	0.6192
Other	333 (8.3%)	277 (8.57%)	56 (7.19%)	0.4044
Total	4011 (100.00%)	3232 (100.00%)	779 (100.00%)	-

**Table 4:** Age-Stratified Distribution of Diagnostic Categories among Adult Liver Transplant Recipients.

Diagnosis Group	18–35 Years Old	36–50 Years Old	51–65 Years Old	65+ Years Old	Total	*p*
Infections	112 (16.69%)	330 (38.28%)	638 (43.82%)	76 (31.28%)	1156 (35.77%)	<0.001
Cryptogenic	117 (17.44%)	183 (21.23%)	419 (28.78%)	80 (32.92%)	799 (24.72%)	<0.001
Toxic	103 (15.35%)	79 (9.16%)	95 (6.52%)	20 (8.23%)	297 (9.19%)	<0.001
Malignancy	59 (8.79%)	59 (6.84%)	124 (8.52%)	36 (14.81%)	278 (8.60%)	0.005
Metabolic	61 (9.09%)	18 (2.09%)	12 (0.82%)	3 (1.23%)	94 (2.91%)	<0.001
Bile Duct Diseases	6 (0.89%)	5 (0.58%)	5 (0.34%)	5 (2.06%)	21 (0.65%)	0.003
Autoimmune Disorder	54 (8.05%)	59 (6.84%)	59 (4.05%)	11 (4.53%)	183 (5.66%)	0.001
Vascular	81 (12.07%)	35 (4.06%)	11 (0.76%)	0 (0.00%)	127 (3.93%)	<0.001
Other	78 (11.62%)	94 (10.90%)	93 (6.39%)	12 (4.94%)	277 (8.57%)	<0.001
Total	671 (100%)	862 (100%)	1456 (100%)	243 (100%)	3232 (100%)	<0.001

**Table 5:** Comparative Analysis of Liver Disease Distribution Between Adult Age Groups: Odds Ratios and *p*-values.

Diagnosis Group	18–35 vs. 36–50	18–35 vs. 51–65	18–35 vs. 65+	36–50 vs. 51–65	36–50 vs. 65+	51–65 vs. 65+	*p*
Infections	3.10 (0.009)	3.89 (0.007)	2.27 (0.016)	1.26 (0.876)	0.73 (0.752)	0.58 (0.038)	<0.001
Cryptogenic	1.28 (0.411)	1.91 (0.023)	2.32 (0.015)	1.50 (0.187)	1.82 (0.029)	1.21 (0.736)	<0.001
Toxic	0.56 (0.034)	0.38 (0.017)	0.49 (0.026)	0.70 (0.041)	0.89 (0.634)	1.28 (0.725)	<0.001
Malignancy	0.76 (0.509)	0.97 (0.900)	1.80 (0.034)	1.27 (0.633)	2.37 (0.014)	1.87 (0.035)	0.005
Metabolic	0.21 (0.012)	0.08 (0.007)	0.13 (0.009)	0.39 (0.025)	0.59 (0.042)	1.50 (0.634)	<0.001
Bile Duct Diseases	0.65 (0.050)	0.38 (0.050)	2.33 (0.050)	0.58 (0.050)	3.59 (0.036)	6.15 (0.005)	0.003
Immune Disorder	0.84 (0.999)	0.48 (0.034)	0.54 (0.043)	0.58 (0.047)	0.65 (0.634)	1.12 (0.867)	0.001
Thrombus	0.31 (0.015)	0.06 (0.005)	0.01 (0.003)	0.18 (0.012)	0.01 (0.001)	0.01 (0.001)	<0.001
Other	0.93 (0.999)	0.52 (0.033)	0.39 (0.020)	0.56 (0.049)	0.43 (0.021)	0.76 (0.678)	<0.001

**Table 6:** Age-Stratified Distribution of Diagnostic Categories among Pediatric Liver Transplant Recipients.

Diagnosis Group	0–2 Years Old	3–7 Years Old	8–12 Years Old	13–17 Years Old	Toplam	*p*
Infections	3 (1.01%)	1 (0.48%)	1 (0.70%)	1 (0.76%)	6 (0.77%)	0.856
Cryptogenic	22 (7.41%)	26 (12.44%)	25 (17.61%)	28 (21.37%)	101 (12.97%)	<0.001
Toxic	61 (20.54%)	88 (42.11%)	54 (38.03%)	26 (19.85%)	229 (29.40%)	<0.001
Malignancy	9 (3.03%)	9 (4.31%)	7 (4.93%)	1 (0.76%)	26 (3.34%)	0.041
Metabolic	72 (24.24%)	40 (19.14%)	28 (19.72%)	36 (27.48%)	176 (22.59%)	0.168
Bile Duct Diseases	101 (34.01%)	12 (5.74%)	7 (4.93%)	4 (3.05%)	124 (15.92%)	<0.001
Immune Disorder	4 (1.35%)	4 (1.91%)	9 (6.34%)	17 (12.98%)	34 (4.36%)	<0.001
Thrombus	3 (1.01%)	11 (5.26%)	3 (2.11%)	10 (7.63%)	27 (3.47%)	<0.001
Other	22 (7.41%)	18 (8.61%)	8 (5.63%)	8 (6.11%)	56 (7.19%)	0.726
Total	297 (100%)	209 (100%)	142 (100%)	131 (100%)	779 (100%)	<0.001

**Table 7:** Comparative Analysis of Liver Disease Distribution Between Pediatric Age Groups: Odds Ratios and *p*-values.

Diagnosis Group	0–2 vs. 3–7	0–2 vs. 8–12	0–2 vs. 13–17	3–7 vs. 8–12	3–7 vs. 13–17	8–12 vs. 13–17	*p*
Infections	0.47 (0.489)	0.70 (0.762)	0.75 (0.801)	1.47 (0.784)	1.60 (0.746)	1.08 (0.958)	0.856
Cryptogenic	1.77 (0.045)	2.67 (0.001)	3.40 (0.001)	1.50 (0.181)	1.92 (0.023)	1.27 (0.414)	<0.001
Toxic	2.81 (0.001)	2.38 (0.001)	0.96 (0.872)	0.84 (0.418)	0.34 (0.001)	0.40 (0.001)	<0.001
Malignancy	1.44 (0.435)	1.66 (0.303)	0.25 (0.137)	1.15 (0.782)	0.17 (0.048)	0.15 (0.046)	0.041
Metabolic	0.74 (0.171)	0.77 (0.295)	1.19 (0.481)	1.04 (0.893)	1.60 (0.047)	1.54 (0.042)	0.168
Bile Duct Diseases	0.12 (0.001)	0.10 (0.001)	0.06 (0.001)	0.85 (0.752)	0.52 (0.265)	0.61 (0.437)	<0.001
Immune Disorder	1.43 (0.613)	4.96 (0.004)	10.92 (0.001)	3.47 (0.027)	7.65 (0.001)	2.20 (0.058)	<0.001
Thrombus	5.44 (0.005)	2.12 (0.349)	8.11 (0.001)	0.39 (0.045)	1.49 (0.368)	3.82 (0.019)	<0.001
Other	1.18 (0.620)	0.75 (0.491)	0.81 (0.632)	0.63 (0.289)	0.69 (0.391)	1.09 (0.858)	0.726

**Table 8:** Distribution of Blood Groups among Donors and Recipients.

Blood Group	Donors	Recipients
O+	1414 (35.29)	850 (21.23)
O−	289 (7.20)	49 (1.22)
A+	1054 (26.26)	1389 (34.61)
A−	291 (7.25)	174 (4.34)
B+	497 (12.38)	720 (17.94)
B−	134 (3.34)	78 (1.94)
AB+	270 (6.73)	675 (16.82)
AB−	62 (1.54)	76 (1.89)
Total	4011 (100.00)	4011 (100.00)

**Table 9:** Frequency and Proportion of Donor-Recipient Blood Group Matches: A Cross-Tabulation Analysis.

	Recipients								
Donors	O+	O−	A+	A−	B+	B−	AB+	AB−	Total
O+	758 (18.9)	0 (0.0)	331 (8.2)	0 (0.0)	221 (5.5)	0 (0.0)	104 (2.6)	0 (0.0)	1414 (35.3)
O−	92 (2.3)	49 (1.2)	47 (1.2)	27 (0.7)	31 (0.8)	18 (0.4)	15 (0.4)	10 (0.2)	289 (7.2)
A+	0 (0.0)	0 (0.0)	907 (22.6)	0 (0.0)	0 (0.0)	0 (0.0)	147 (3.7)	0 (0.0)	1054 (26.3)
A−	0 (0.0)	0 (0.0)	104 (2.6)	147 (3.7)	0 (0.0)	0 (0.0)	18 (0.4)	22 (0.5)	291 (7.3)
B+	0 (0.0)	0 (0.0)	0 (0.0)	0 (0.0)	417 (10.4)	0 (0.0)	80 (2.0)	0 (0.0)	497 (12.4)
B−	0 (0.0)	0 (0.0)	0 (0.0)	0 (0.0)	51 (1.3)	60 (1.5)	10 (0.2)	13 (0.3)	134 (3.3)
AB+	0 (0.0)	0 (0.0)	0 (0.0)	0 (0.0)	0 (0.0)	0 (0.0)	270 (6.7)	0 (0.0)	270 (6.7)
AB−	0 (0.0)	0 (0.0)	0 (0.0)	0 (0.0)	0 (0.0)	0 (0.0)	31 (0.8)	31 (0.8)	62 (1.5)
Total	850 (21.2)	49 (1.2)	1389 (34.6)	174 (4.3)	720 (17.9)	78 (1.9)	675 (16.8)	76 (1.9)	4011 (100.0)

**Table 10:** Demographic Characteristics of Liver Donors.

Feature	Number ± SD	% or Median [Range]
Gender (M/F, %)	2383/1594	59.9%/40.1%
General Age	31.9 ± 11.0	30 [0–76]
Adult/Pediatric Donor	3911/95	97.6%/2.4%
Adult Age	32.5 ± 10.4	30 [18–76]
Adult Gender (M/F, %)	2326/1556	59.9%/40.1%
Pediatric Age	7.7 ± 5.9	6 [0–18]
Pediatric Gender (M/F, %)	57/38	60.0%/40.0%
Cadaver/Living Donor (%)	530/3481	13.2%/86.8%

**Table 11:** Demographic and Kinship Characteristics of Living Liver Donors.

Feature		Number ± SD	% or Median [Range]
Total Live Donors		3481	100%
Gender (M/F, %)		2058/1399	59.5%/40.5%
Age		30.8 ± 8.8	29 [0–63]
The Most Common Kinship Relationships	Son	746	21.4%
	Brother	586	16.8%
	Unrelated	489	14.0%
	Daughter	425	12.2%
	Cross transplant	234	6.7%
	Mother	224	6.4%
	Spouse	162	4.7%
	Father	138	4.0%

**Table 12:** Donor Relative Relationships of Recipients Under and Over 18 Years of Age.

Donor Kinship	Adults	Pediatrics	*p* Value
Mother	188 (24.04)	39 (1.21)	<0.01
Father	126 (16.20)	12 (0.37)	<0.001
Cadaver	156 (20.05)	377 (11.66)	<0.1
Unrelated	145 (18.64)	344 (10.64)	<0.1
Brother	49 (6.30)	541 (16.74)	<0.1
Child (Son)	0 (0.00)	751 (23.24)	<0.0001
Child (Girl)	0 (0.00)	430 (13.30)	<0.0001
Cross Transfer	39 (5.01)	195 (6.03)	0.288
Nephew	0 (0.00)	179 (5.54)	<0.0001
Spouse	0 (0.00)	168 (5.20)	<0.0001
Cousin	18 (2.31)	117 (3.62)	0.08
Uncle	27 (3.47)	8 (0.25)	<0.05
Uncle	13 (1.67)	6 (0.19)	<0.05
Grandchild	0 (0.00)	17 (0.53)	<0.0001
Daughter-in-law	0 (0.00)	16 (0.50)	<0.0001
Still	10 (1.29)	2 (0.06)	<0.01
Auntie	8 (1.03)	3 (0.09)	<0.05
Relationship in Law	0 (0.00)	7 (0.22)	<0.0001
Sister-in-law	0 (0.00)	6 (0.19)	<0.0001
Groom	0 (0.00)	6 (0.19)	<0.0001
Sister-in-law	0 (0.00)	5 (0.15)	<0.0001
Brother-in-law	0 (0.00)	2 (0.06)	<0.0001
Brother-in-law	0 (0.00)	1 (0.03)	<0.0001
TOTAL	779 (100.00)	3232 (100.00)	

**Table 13:** Geographic Distribution of Pediatric and Adult Liver Recipients.

	Pediatric Patients		Adult Patients	
Order	Home Town	Percentage (%)	Home Town	Percentage (%)
1	Abroad (Syria)	20	Malatya	8.57
2	Sanliurfa	12.07	Diyarbakir	8.14
3	Gaziantep	8.60	Sanliurfa	7.04
4	Malatya	6.29	Kahramanmaras	5.42
5	Diyarbakir	6.16	Gaziantep	5.17
6	Kahramanmaras	5.39	Abroad (Syria)	4.52
7	Adiyaman	3.08	Elazig	4.52
8	Adana	3.08	Adiyaman	4.40
9	Mardin	2.70	Adana	3.71
10	Kayseri	2.44	Kayseri	3.37

**Table 14:** Central Distribution of Cadaver Donors.

Order	Cadaver Center	Percentage (%)
1	Marmara Region	15.6
2	Aegean Region	18.1
3	Eastern Anatolia Region	17.5
4	Mediterranean Region	19.6
5	Central Anatolia Region	11.8
6	Black Sea Region	8.4
7	Southern Marmara Region	4.3
8	Southeastern Anatolia Region	4.3

**Table 15:** Liver Transplantation Numbers by Year.

Year	Transplant Number	%
2002	3	0.07%
2003	1	0.02%
2004	6	0.15%
2005	1	0.02%
2006	11	0.27%
2007	54	1.35%
2008	153	3.81%
2009	108	2.69%
2010	175	4.36%
2011	222	5.53%
2012	185	4.61%
2013	304	7.58%
2014	219	5.46%
2015	203	5.06%
2016	229	5.71%
2017	223	5.56%
2018	272	6.78%
2019	277	6.91%
2020	244	6.08%
2021	231	5.76%
2022	280	6.98%
2023	240	5.98%
2024	281	7.01%
2025	89	2.22%
Total	4011	100.00%

## Data Availability

The data presented in this study are available on request from the corresponding author due to privacy restrictions.

## Abbreviations

The following abbreviations are used in this manuscript:

LDLT	Living Donor Liver Transplantation
LPE	Liver Paired Exchange
